# Factors affecting the STR amplification success in poorly preserved bone samples

**DOI:** 10.1186/2041-2223-1-9

**Published:** 2010-10-04

**Authors:** Mikko T Putkonen, Jukka U Palo, Jose M Cano, Minttu Hedman, Antti Sajantila

**Affiliations:** 1 Department of Forensic Medicine, Hjelt Institute, University of Helsinki, PO Box 40, 00014 University of Helsinki, Helsinki, Finland; 2 Department of Biosciences, University of Helsinki, Helsinki, Finland

## Abstract

**Background:**

Factors affecting the success of short tandem repeat (STR) amplification of poorly preserved samples are generally known, but as of yet, they have seldom been systematically assessed. Using two different maximum likelihood-based methods, the relative importance of DNA quantity, degradation and inhibition in STR genotyping was studied with DNA extracts from a set of old bone samples. First, the effects of different factors related to PCR amplification were estimated with a generalized linear mixed model. Second, error rates of allelic drop-out and drop-in were estimated on the basis of the frequency and nature of mismatches between replicates.

**Results:**

In autosomal STR analyses, the most important factor was the DNA quantity, followed by the degradation, whereas in Y-chromosomal STR analysis, the most important factor was the degradation. Inhibition was a minor concern in STR analyses of poorly preserved bones.

**Conclusions:**

The success of PCR amplification depends largely on the template DNA quality (amount and degradation), but these problems can be partly compensated for by different primer design and amplification chemistry. Consequently, the relative roles of the compromising factors differ according to the kit used.

## Background

Geneticists working in the fields of forensics and ancient DNA are frequently forced to attempt the recovery of amplifiable DNA from quantificatively and qualitatively suboptimal material. Successful PCR amplification from old and/or otherwise poorly preserved specimens is challenging, because the success depends on several factors, such as the amount of recoverable DNA, the level of DNA damage and the inhibiting agents present. In addition, the chemistry and methods used for DNA extraction and amplification may have a strong effect on the amplification success. The role of these different attributes has been recognized [1-3], but their relative significance has rarely been assessed.

In this study, the roles of the compromising factors were evaluated by investigating the effect on PCR success of both amount and quality of the template DNA, and of the amplification kit used. To accomplish this goal we estimated the relative roles of various factors simultaneously using univariate variance analysis. Owing to the stochastic effects associated with the analyses of samples of low DNA quantity, evaluation of the compromising factors is important in increasing the reliability and efficiency of the analysis [4-6]. Recently, there has been a dramatic increase in the number attempts of recovering DNA from poor quality samples, such as archaeological and forensic bones [6-8].

## Materials and methods

DNA extracts of poorly preserved bone samples were genotyped with three different commercial short tandem repeat (STR) kits. The PCR success, measured by the relative fluorescence unit (RFU) values, was evaluated in relation to various attributes: amount of DNA present in the extracts, inhibition (as determined by the quantification assay), amplicon length and STR kit used. This allowed correlation of the effects of DNA quantity, inhibitors, degradation and PCR chemistry (for example, primers) to PCR success. The interdependence of PCR success and size of the amplified PCR product (allele size) was used to assess degradation of the template DNA. In addition, the number of stochastic genotyping errors (allelic drop-out and drop-in rates) was estimated.

### Extraction, quantification of DNA and assessment of inhibition

Human bones around 70 years old were used as the material for the study. These bones had been recovered from 14 different locations (each with several excavation sites) in an area of about 100 × 200 km for identification of missing Finnish World War II soldiers [9]. These human remains were recovered from different depths and soil types in southern boreal forests. The bones had been subjected to various taphonomic factors, which had resulted in differing levels of physical degradation.

DNA was extracted from femoral bones of 45 individuals. Before DNA extraction, the bone surfaces were cleansed mechanically using sterile toothbrushes, then rinsed once with sterile water and once with sodium hypochlorite solution containing 0.08% active chlorine. Finally, the bones were rinsed with 70% ethanol and dried at room temperature for a minimum of 24 hours. Using a dental hand drill (Faro, Milan, Italy) the bone surface was stripped, and the revealed inner compact bone tissue pulverized. Approximately 0.5 g of bone powder was incubated in lysis buffer at 56°C overnight according to a previously described protocol [10]. DNA was extracted from the lysates by the standard phenol-chloroform-isoamyl alcohol method [11] on 50 ml phase-lock gel columns (5 Prime, Hamburg, Germany) and concentrated by centrifugation, reducing the total volume to 150 to 200 μl, on 50 ml columns (Amicon Ultra 30; Millipore, Billerica, MA USA). Finally, the extracts were purified (QIAQuick PCR Purification Kit; Qiagen, Hilden, Germany).

Extractions were performed with strict precautions including protective clothing, with equipment and surfaces treated with chlorine and/or irradiated with UV. Only a small number (≤ 6) of bone samples were extracted at one time, and blank controls were used to detect possible contamination. Furthermore, the genotyping results were compared against the lab personnel profiles to check for authenticity.

Nuclear DNA and inhibition were quantified on a real-time PCR system (ABI7500; Applied Biosystems, Foster City, CA USA) using a quantification kit (Quantifiler™Human DNA Quantification Kit; Applied Biosystems) according to the manufacturer's recommendations. Quantification of the mitochondrial (mt)DNA was performed as described previously [12], with two amplicons (102 and 143 bp) allowing crude estimation of degradation.

### Genotyping and assessment of PCR chemistry

Y-chromosomal and autosomal STR loci were amplified from two parallel 1 μl aliquots of extracted DNA samples using three different amplifier kits (AmpF*l*STR^® ^Yfiler™(YF), AmpF*l*STR^® ^Minifiler™(MF) and AmpF*l*STR^® ^Identifiler™(IF); all Applied Biosystems). YF amplifies 17 Y-chromosomal STRs with allele sizes ranging from 103 to 291 bp. MF and IF amplify a sex-specific amelogenin locus (107 and 113 bp alleles) together with eight and 15 autosomal loci with allele sizes ranging from 96 to 216 bp and 125 to 315 bp, respectively. PCR was performed in a thermal cycler (Tetrad PTC-225; MJ Research, Waltham, MA USA) according to the manufacturer's instructions, apart from the number of PCR cycles (32 for Y-chromosome STRs, 30 for autosomal STRs) and the reaction volume (12.5 μl). Positive (male DNA, catalogue number 9947A or 9948; Promega Corp., Sunnyvale, CA USA) and negative (water filtered through Milli-Q filters; Millipore) controls were included in all analyses. The PCR products were analyzed with capillary electrophoresis (ABI Prism^® ^310; Applied Biosystems) using 10 second and 15.0 kV injection.

The genotyping results were analyzed (GeneMapperID™V.3.2; Applied Biosystems), using both 30 and 100 RFU as the limit of detection in the allele peak scoring. Hereafter, the combination of the kit and the limit of detection are abbreviated as IF30, IF100, MF30, MF100, YF30 and YF100. For the peak height imbalance, a relaxed criterion of smaller peak being ≥ 30% of the larger peak height was adopted. For each peak, the RFU value was used as an indicator of amplification success in the subsequent data analyses.

### Evaluation of STR amplification in relation to compromising factors

A maximum likelihood (ML) based generalized linear mixed model (GLMM), using the PROC MIXED procedure and the COVTEST statement implemented in SAS software V.9.1 (SAS Institute Inc., Cary, NC USA) was used to estimate the effects of allele size, quantity of DNA, inhibition, and amplification kit (modelled as fixed effects) on the PCR success (RFU values), while accounting for any covariation inherent to the structure of the data; that is, individual sample, replicate (nested in individual), locus and allele (first or second allele nested in a locus), modelled as random effects.

The model was run separately for a nSTR dataset of each studied kit with both limits of detection (30 and 100 RFU). For assessing the effect of the kit, datasets consisting of nine common loci in IF and MF were pooled and analyzed together with both limits of detection.

Allelic drop-out and drop-in rates for the autosomal data were assessed as an additional indicator of the PCR amplification success. The frequencies of these stochastic genotyping errors were estimated with a method implemented in the software Pedant V.1.0 [13]. The 95% confidence intervals for these estimates were determined by 10,000 randomization steps. The method is based on ML estimation of the drop-out and drop-in rates from the frequency and nature of mismatches between two replicate amplifications. As a given parameter value for each locus, the expected heterozygosity (H_e_), ranging from 0.59 to 0.87, was estimated from a random sample of 200 Finnish people.

## Results and Discussion

The main picture emerging from this study is that the two most important factors affecting PCR success are DNA quantity and degradation. Inhibition was found to be relatively insignificant. All the GLMM analyses were made separately for limits of detection set to 30 and 100 RFU, but both gave similar results (not shown). Consequently, the discussion is mainly based on the results with the limit of detection of 100 RFU.

Overall, the GLMM analysis showed that PCR success was significantly affected by DNA quantity, allele size and amplification kit (see Additional file 1). DNA quantity had a larger effect on the MF amplification kit, and inhibition was also found to affect only the MF kit (see Additional file 1). Variance in RFU values was explained in part by sample (average across kits and range: 11% (range 0.5 to 21%), 0.0001 ≤ *P *≤ 0.384), replicate (3.5% (1.0 to 5.0%), 0.001 ≤ *P *≤ 0.070), locus (21% (16 to 28%), 0.004 ≤ *P *≤ 0.028), but mostly by residual variance, (66.5% (58 to 79%), *P *< 0.0001). The residual variance is the variance in RFU values not accounted by the factors (that is, fixed and random) included in the model.

The validity of the data was also assessed by estimating the drop-in rates in autosomal STR analyses. The point estimate of drop-ins was 0.01 (95% CI, 0.00 - 0.02) in all autosomal STR analyses, which was close to the estimated contamination level reported previously of 0.008 ± 0.002% [14] and 1.36% [15], supporting the validity of the analysis results, even with the limit of detection set as low as 30 RFU.

### DNA quantity

The quantity of nuclear DNA per sample varied from 'undetectable' (five samples) to 1.5 ng/μl, with an average of 0.47 ng/μl (95% CI, 0.46 - 0.48). According to the manufacturer's sensitivity studies, the nuclear DNA quantification assay can detect DNA concentrations of 0.016 ng/μl. The DNA quantity was the most important factor in PCR success for autosomal STR data, and the second most important factor in the Y-chromosomal data (see Additional file 1, *F *values).

For each amplification reaction, 1 μl of DNA extract was used, corresponding to an estimated quantity of 0 to 1.5 ng nuclear DNA per reaction. Reproducibility of the PCR amplifications was comparable with the template concentration; however, the samples with the lowest detected DNA concentration (0.04 ng/μl) also yielded some reproducible amplification products. Four of the five samples with 'undetectable' nuclear DNA concentration also exhibited 100% inhibition.

The quantities of mtDNA did not appear to have a significant effect on the success of STR amplification (that is, RFU values) (see Additional file 1). This is in line with previous studies, such as that of Schwarz *et al. *[16], which showed highly variable ratio of mtDNA to nuclear DNA in mammoth (*Mammuthus primigenius*) DNA samples. In the casework, successful DNA sequences in the mitochondrial HVR1 (nucleotide positions 16024 - 16385) and HVR2 (72 - 340) regions were obtained from 40 of the samples. For the remaining five samples, the sequences were obtained after additional laboratory work.

### Degradation

Rather than DNA quantity, allele size was the most important contributor to the RFU value in the Y-chromosomal STR data. The effect of allele size was 1.4 times the effect of the quantity of nuclear DNA (see Additional file 1, *F *values). In autosomal STRs, the corresponding effect was 0.7 in IF analysis and 0.1 times in MF analysis.

**Figure 1:**
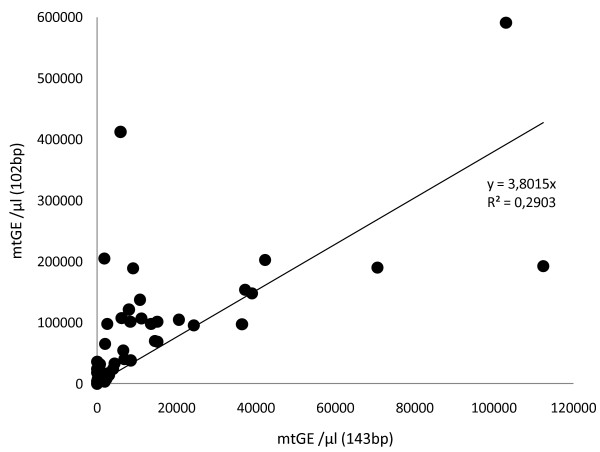
**Regression between quantities of mitochondrial genomes (mtGE) measured with amplicons 102 bp and 143 bp in length**. The intercept is forced to origin. *R^2 ^*is the coefficient of determination of linear regression.

The mtDNA quantification results also yielded information about degradation. The quantities determined with the 102 bp amplicon were around 3.8 times greater than the quantities with the 143 bp amplicon, implicating degradation of (mt)DNA (Figure 1). It seems that mtDNA and nuclear DNA degrade at varying relative rates under different conditions, as has been reported previously [16].

**Figure 2:**
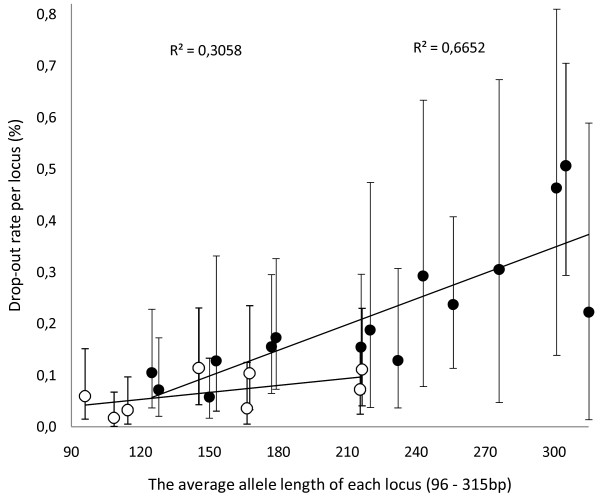
**Dropout rates in autosomal short tandem repeat (STR) analyses**. IF (black circles) and MF (white circles), plotted against the average allele length of each locus. Limit of detection was 100 relative fluorescence units (RFU).

The effect of degradation on autosomal STRs was assessed by an allelic drop-out study. Average estimates of drop-out frequencies were 0.07 (95% CI, 0.04 - 0.10) for MF loci and 0.20 (95% CI, 0.13 - 0.27) for IF loci. In the shortest IF loci (average allele sizes ranging from 125 to 216 bp) the average drop-out frequency 0.11 (95% CI, 0.07 - 0.15) was similar to that of MF loci, indicating that the effect stems largely from the template DNA degradation (Figure 2). The connection between decreased amplifiability of longer amplicons and degradation of DNA is not a new finding [17-19], and it is intuitively evident. However, care is needed when interpreting degradation from the amplification differences of independent loci, because each locus is amplified with a different set of primers. High interlocus variance (16 to 28%) in RFU values can be seen as implying differences in amplification chemistry. Nevertheless, the results of quantification of mtDNA at the same locus with amplicons of different lengths as well as the estimates of the drop-out rates support the notion of degradation.

The estimated drop-out rates are unacceptably high for routine work, but are here explained largely by the unmodified experimental setup, in which DNA quantity was taken as an independent variable. The PCR amplifications were also performed without adjusting template concentrations or successive optimization of conditions.

When considering the effect of amplicon length on PCR success, the effect of buffer and/or enzyme differences between different kits cannot be completely ignored. However, similar drop-out frequencies observed in shorter amplicons of different kits support the conclusion that instead of the differences in the amplification chemistries, the degradation (amplicon length), is decisive. However, similar drop-out frequencies observed in shorter amplicons with different kits support the conclusion that it is the degradation (the amplicon length) rather than the differences in the amplification chemistries that is decisive. In regression analysis of drop-out rates and amplicon sizes, the only significant regression was observed in IF data (see Additional file 2). This is probably a result of the large size range of the amplicons in the IF data contributing to the increased differences within the dataset.

The ML estimates for drop-out and drop-in rates are only obtainable for diploid data. At the Y-chromosomal loci, 67% of the samples yielded an amplification product in both replicates, 13% of the samples yielded a full profile, and in 46% of the samples, at least 13 out of 17 loci amplified.

### Inhibition

Inhibition estimates varied from 0% (7 samples) to 100% (6 samples), with an average of 15% and median of 3%. The amount of inhibition had a bimodal distribution around 0 to 25% and 100%.

In the MF data, inhibition was a significant factor, the effect of which was around three times that of the allele size (see Additional file 1, *F *values). This does not necessarily mean poor performance in absolute terms. The shorter amplicons are less sensitive to degradation, which is likely to increase the relative importance of inhibition. When the relaxed limit of detection of 30 was applied, the effect increased to around nine times the effect of the allele size (not shown). By contrast, inhibition had little effect on YF data (see Additional file 1). Even in the samples with an estimated 100% inhibition, on average of 8.0 loci could be amplified, and one of the samples yielded 15 scorable loci. No significant regression between the estimated inhibition level and amount of amplified loci was found with any amplification method. However, it is worth noting that the experiment setup was not designed to answer this question specifically.

### Amplification kit

**Figure 3:**
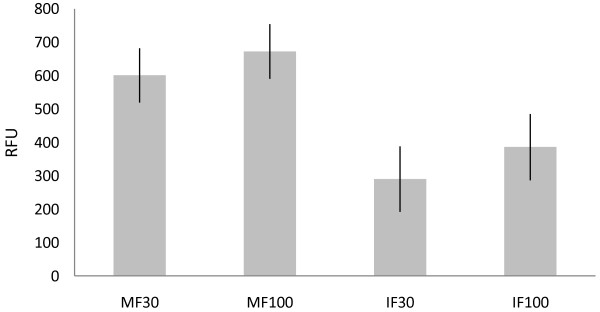
**Least squares means of relative fluorescence unit (RFU) values for the nine loci common for both AmpFlSTR^® ^Minifiler™(MF) and AmpFlSTR^® ^Identifiler™(IF) kits**. Bars represent 95% confidence intervals in both limits of detection (30 and 100 RFU)

Autosomal STR chemistry (IF or MF) had a significant effect on PCR success. The effect was about 0.5 times the effect of DNA quantity and 2.1 times the effect of inhibition (see Additional file 1, *F *values). When the relaxed limit of detection (30 RFU) was used, the effect of kit rose to the level of DNA quantity and up to 4.9 times the effect of inhibition (not shown). Here, the relative increase in the kit effect resulted from a stronger bias to shorter amplicons (that is, MF data) with the relaxed limit of detection of 30 RFU. In the different kits, different primers are used to amplify the same loci, and in poorly preserved samples shorter amplicons surpass the longer ones in amplification efficiency. This has also been the aim in the introduction of mini-STR kits. The differences between kits are illustrated in Figure 3, showing the least square means of the RFUs of both kits with 95% CI.

## Conclusions

The quantity of nuclear DNA quantity and its degradation (allele size) were the most significant factors affecting PCR success from poorly preserved bones. Compared with the effect of DNA amount and degradation, inhibition is a minor concern, but its relative role increases when shorter amplicons are amplified.

This study highlights the requirement for efficient DNA extraction. A number of different methods have been developed for DNA extraction, with varying performances from different types of starting material [17,20,21]. The extraction method in the present study was fixed, and was based on the protocol validated in our laboratory. DNA analyses from suboptimal material require laboratory-specific method adjustment and validation for each sample type. The most significant problems, such as degradation, are template-dependent and cannot be completely circumvented. Nevertheless, by improving the PCR (for example, by short amplicons, with modifications of the buffer system or enzymes used) these problems can be partially overcome. This is supported by the current results, showing that the existing PCR amplification chemistries were affected differently by the compromising factors.

## Competing interests

The authors declare that they have no competing interests.

## Authors' contributions

MTP, JUP and AS designed the study. MTP carried out the laboratory work. JMC and MTP performed the statistical tests. All the authors contributed to the writing of the manuscript, and read and approved the final manuscript.

## Supplementary Material

Additional file 1 **Fixed effect (DNA Quantity, inhibition, allele size and kit) results from the GLMM analyses in different analyses with limit of detection being 100 relative fluorescence units (RFU)**. Effect size of each factor is given as *F *value, which denotes the difference between the overall mean of the RFU values and the mean RFU for each factor. Significant *P *values are in bold. DNAQ = nuclear DNA quantity, DNAQ_S and DNA_L = mtDNA quantity (102 bp and 143 bp fragments respectively). Click here for file

Additional file 2 **Regression analysis of drop-out rates in different autosomal short tandem repeat (STR) analyses analysed with the software Pedant V.1.0**. The effect sizes are given as *F *values. Significant *P *values are in bold. For abbreviations, see text. Click here for file

## References

[B1] Opel KL Chung D McCord BR A study of PCR inhibition mechanisms using real time PCR J Forensic Sci 2010 55 25 33 10.1111/j.1556-4029.2009.01245.x 20015162

[B2] Kontanis EJ Reed FA Evaluation of real-time PCR amplification efficiencies to detect PCR inhibitors J Forensic Sci 2006 51 795 804 10.1111/j.1556-4029.2006.00182.x 16882221

[B3] Wilson IG Inhibition and facilitation of nucleic acid amplification Appl Environ Microbiol 1997 63 3741 3751 9327537 10.1128/aem.63.10.3741-3751.1997 PMC168683

[B4] Caragine T Mikulasovich R Tamariz J Bajda E Sebestyen J Baum H Prinz M Validation of testing and interpretation protocols for low template DNA samples using AmpFlSTR^® ^Identifiler^®^ Croat Med J 2009 50 250 267 10.3325/cmj.2009.50.250 19480021 PMC2702740

[B5] Julio JM Chien Wei C Robert EL Dennis YW Jennifer LB Timothy PM Lori KH Development and validation of the development and validation of the AmpFlSTR^® ^MiniFilerTM PCR amplification kit: A miniSTR multiplex for the analysis of degraded and/or PCR inhibited DNA J Forensic Sci 2008 53 838 852 10.1111/j.1556-4029.2008.00760.x 18540972

[B6] Malmström H Gilbert MTP Thomas MG Brandström M Storå J Molnar P Andersen PK Bendixen C Holmlund G Götherström A Willerslev E Ancient DNA reveals lack of continuity between Neolithic hunter-gatherers and contemporary Scandinavians Curr Biol 2009 19 1758 1762 10.1016/j.cub.2009.09.017 19781941

[B7] Coble MD Loreille OM Wadhams MJ Edson SM Maynard K Meyer CE Niederstätter H Berger C Berger B Falsetti AB Gill P Parson W Finelli LN Mystery solved: the identification of the two missing Romanov children using DNA analysis PLoS ONE 2009 4 e4838 10.1371/journal.pone.0004838 19277206 PMC2652717

[B8] Parsons TJ Huel R Davoren J Katzmarzyk C Milos A Selmanovic A Smajlovic L Coble MD Rizvic A Application of novel "mini-amplicon" STR multiplexes to high volume casework on degraded skeletal remains Forensic Sci Int Genet 2007 1 175 179 10.1016/j.fsigen.2007.02.003 19083751

[B9] Palo JU Hedman M Söderholm N Sajantila A Repatriation and identification of Finnish World War II soldiers Croat Med J 2007 48 528 535 17696308 PMC2080560

[B10] Loreille OM Diegoli TM Irwin JA Coble MD Parsons TJ High efficiency DNA extraction from bone by total demineralization Forensic Sci Int Genet 2007 1 191 195 10.1016/j.fsigen.2007.02.006 19083754

[B11] Sambrook J Fritsch EF Maniatis T Molecular Cloning. A Laboratory Manual 1989 2 New York: Cold Spring Harbor Laboratory Press

[B12] Niederstätter H Köchl S Grubwieser P Pavlic M Steinlechner M Parson W A modular real-time PCR concept for determining the quantity and quality of human nuclear and mitochondrial DNA Forensic Sci Int Genet 2007 1 29 34 10.1016/j.fsigen.2006.10.007 19083725

[B13] Johnson PCD Haydon DT Maximum-likelihood estimation of allelic dropout and false allele error rates from microsatellite genotypes in the absence of reference data Genetics 2007 175 827 842 10.1534/genetics.106.064618 17179070 PMC1800638

[B14] Rasmussen M Li Y Lindgreen S Pedersen JS Albrechtsen A Moltke I Metspalu M Metspalu E Kivisild T Gupta R Bertalan M Nielsen K Gilbert MT Wang Y Raghavan M Campos PF Kamp HM Wilson AS Gledhill A Tridico S Bunce M Lorenzen ED Binladen J Guo X Zhao J Zhang X Zhang H Li Z Chen M Orlando L Kristiansen K Bak M Tommerup N Bendixen C Pierre TL Grønnow B Meldgaard M Andreasen C Fedorova SA Osipova LP Higham TF Ramsey CB Hansen TV Nielsen FC Crawford MH Brunak S Sicheritz-Pontén T Villems R Nielsen R Krogh A Wang J Willerslev E Ancient human genome sequence of an extinct Palaeo-Eskimo Nature 463 757 762 10.1038/nature08835 20148029 PMC3951495

[B15] Sturk KA Coble MD Barritt SM Irwin JA Evaluation of modified Yfiler^(TM) ^amplification strategy for compromised samples Croat Med J 2009 50 228 238 10.3325/cmj.2009.50.228 19480019 PMC2702738

[B16] Schwarz C Debruyne R Kuch M McNally E Schwarcz H Aubrey AD Bada J Poinar H New insights from old bones: DNA preservation and degradation in permafrost preserved mammoth remains Nucleic Acids Res 2009 37 3215 3229 10.1093/nar/gkp159 19321502 PMC2691819

[B17] Pääbo S Ancient Dna - extraction, characterization, molecular-cloning, and enzymatic amplification Proc Natl Acad Sci USA 1989 86 1939 1943 10.1073/pnas.86.6.1939 2928314 PMC286820

[B18] Hummel S Schultes T Bramanti B Herrmann B Ancient DNA profiling by megaplex amplications Electrophoresis 1999 20 1717 1721 10.1002/(SICI)1522-2683(19990101)20:8<1717::AID-ELPS1717>3.0.CO;2-D 10435437

[B19] Wandeler P Smith S Morin PA Pettifor RA Funk SM Patterns of nuclear DNA degeneration over time - a case study in historic teeth samples Mol Ecol 2003 12 1087 1093 10.1046/j.1365-294X.2003.01807.x 12753226

[B20] Keyser-Tracqui C Ludes B Methods for the study of ancient DNA Forensic DNA Typing Protocols 2005 253 264 10.1385/1-59259-867-6:253 15570113

[B21] Kolman CJ Tuross N Ancient DNA analysis of human populations Am J Phys Anthropol 2000 111 5 23 10.1002/(SICI)1096-8644(200001)111:1<5::AID-AJPA2>3.0.CO;2-3 10618586

